# *Eleutherine bulbosa* (Mill.) Urb. Bulb: Review of the Pharmacological Activities and Its Prospects for Application

**DOI:** 10.3390/ijms22136747

**Published:** 2021-06-23

**Authors:** Ammar Akram Kamarudin, Nor Hafiza Sayuti, Norazalina Saad, Nor Asma Ab. Razak, Norhaizan Mohd. Esa

**Affiliations:** 1 Natural Medicine and Product Research Laboratory (NaturMeds), Institute of Bioscience, Universiti Putra Malaysia, Serdang 43400, Selangor, Malaysia; ammarakram071@gmail.com (A.A.K.); norhafiza2312@gmail.com (N.H.S.); norasmarazak@upm.edu.my (N.A.A.R.); 2 Laboratory of Cancer Research UPM-MAKNA (CANRES), Institute of Bioscience, Universiti Putra Malaysia, Serdang 43400, Selangor, Malaysia; norazalina@upm.edu.my; 3 Department of Nutrition, Faculty of Medicine and Health Sciences, Universiti Putra Malaysia, Serdang 43400, Selangor, Malaysia

**Keywords:** *E. bulbosa*, Iridaceae, bioactive, pharmacology, industrial applications

## Abstract

Natural product is an excellent candidate for alternative medicine for disease management. The bulb of *E. bulbosa* is one of the notable Iridaceae family with a variety therapeutic potential that is widely cultivated in Southeast Asia. The bulb has been used traditionally among the Dayak community as a folk medicine to treat several diseases like diabetes, breast cancer, nasal congestion, and fertility problems. The bulb is exceptionally rich in phytochemicals like phenolic and flavonoid derivatives, naphthalene, anthraquinone, and naphthoquinone. The electronic database was searched using various keywords, i.e., *E. bulbosa*, *E. americana*, *E. palmifolia*, *E. platifolia*, and others due to the interchangeably used scientific names of different countries. Scientific investigations revealed that various pharmacological activities were recorded from the bulb of *E. bulbosa* including anti-cancer, anti-diabetic, anti-bacterial, anti-fungi, anti-viral, anti-inflammatory, dermatological problems, anti-oxidant, and anti-fertility. The potential application of the bulb in the food industry and in animal nutrition was also discussed to demonstrate its great versatility. This is a compact study and is the first study to review the extensive pharmacological activities of the *E. bulbosa* bulb and its potential applications. The development of innovative food and pharma products from the bulb of *E. bulbosa* is of great interest.

## 1. Introduction

*Bawang Dayak*, *bawang hutan*, or *bawang tiwai* are locally used for *Eleutherine bulbosa* bulb, a herbaceous, perennial flowering plant from the Iridaceae family, broadly cultivated in South America, the African region, and Indonesia [1,2]. It is widely found across sulphuric land in Indonesia, within 600 to 2000 m above the Kalimantan Island sea level [3]. The bulb is famous among the local tribe of Kalimantan, the Dayaks, where it is traditionally used to treat diabetes, breast cancer, hypertension, stroke, sexual disorders, and to enhance breast milk production [3]. It has multiple scientific names which are used interchangeably according to the region, such as *E. americana*, *E. palmifolia*, and *E. platifolia.* Nonetheless, *E. bulbosa* was proposed as an official scientific name by Kew and Missouri Botanic Garden [4]. Morphologically, *E. bulbosa* is an herbaceous, perennial flowering plant with a red bulb that resembles an onion [5]. The length of the leaves is approximately 25 cm, with five to six petals of white flowers arranged at the apex of the foliage [5]. Naphthalene, anthraquinone, and naphthoquinone are the key constituents of the *E. bulbosa* bulb that demonstrated various pharmacological properties such as anti-microbial, anti-inflammatory, anti-hypertension, anti-cancer, anti-diabetic, and anti-melanogenesis activity [1,2]. However, scientific evidence concerning the biological activities and potential use of the bulb of *E. bulbosa* is scarce. Thus, this is the first study highlighting the recent biological reviews of the *E. bulbosa* bulb and its potential applications in the industry.

## 2. Morphological Characteristics

**Figure 1:**
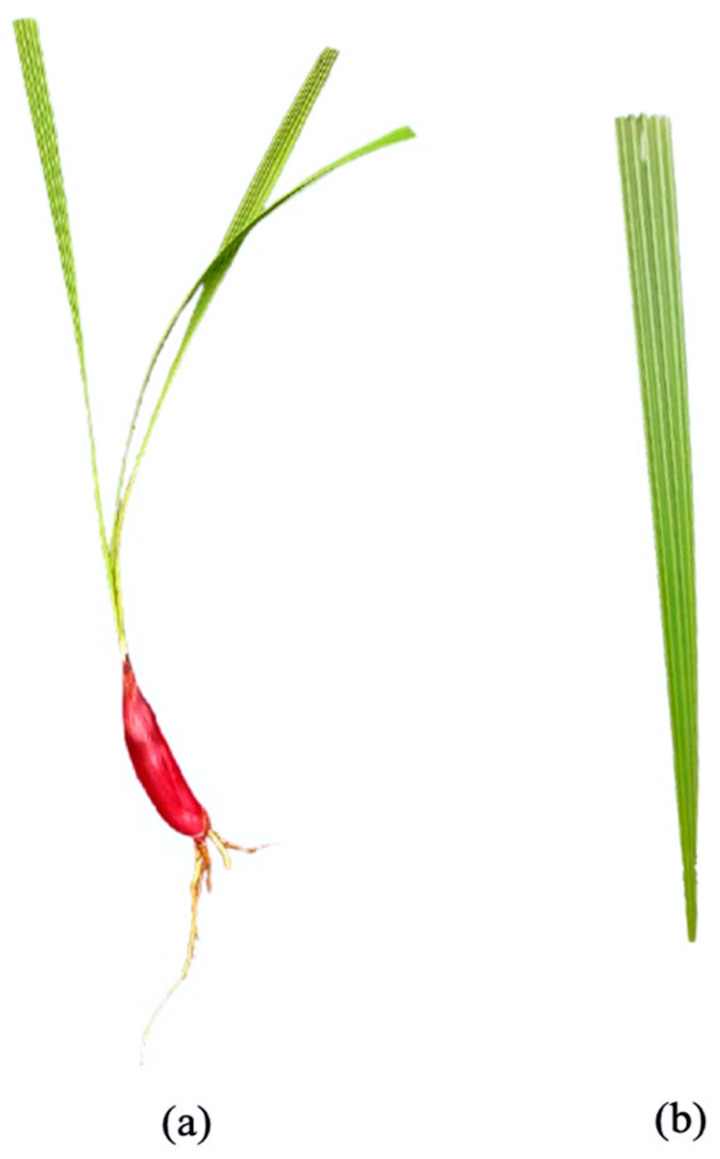
The morphological characteristics of *E. bulbosa* bulb. Note that it is a rhizomatous long, red bulb that nearly mimics the red onion (**a**) and has a leaf in a pleated linear-lanceolate shape (**b**).

The *Eleutherine* genus is described as an herbaceous, rhizomatous, and perennial flowering plant with a predominantly red and wine bulb color similar to red onion (Figure 1), approximately around 20 to 30 cm [4,5]. They have whole leaves, pleated at an average length of 25 cm, with a large panicle of white or pink flowers arranged at the apex of a long, dense scape above the foliage [4]. The *Eleutherine* genus has monosulcate and perforated pollen grain on the proximal surfaces with heterogenous exine at different grain parts [6]. Anatomically, microscopic examination of the *E. americana* bulb demonstrated a different size of parenchymal cells and various shapes of calcium oxalate crystals, predominantly a styloid structure [7]. The bulb is derived from a thick petiole with similar vascular bundles as in the leaves, and it has no sclerenchyma cells for tissue support [7].

## 3. Traditional Uses

The bulb of *E. bulbosa* has been used traditionally since time immemorial. It is used as a carminative agent to relieve flatulence by coordinating and regulating gut contractions [8]. It is also used in combination with *Alpinia galanga* to relieve cold and nasal congestion among children [8]. However, the Dayak tribe specifically uses the bulb for various applications such as diabetic treatment, stroke management, breast cancer, hypertension, and fertility problems, and for enhancing breast-milk production in woman [3]. In other areas, some use it as a treatment for the coronary disorder, abortifacients and emmenagogue, menstrual pain, anti-inflammatory, and wound healing [9,10,11,12,13,14]. Besides that, the bulb of *E. bulbosa* is also traditionally used to treat pertussis, hemoptysis, and simple coughs due to its anti-microbial and anti-inflammatory properties [15]. Treated as a folklore medicine, the juice or chew of the fresh bulb is chewed to manage diarrhea, dysentery, jaundice, colitis, and in some instances, colon cancer [15].

## 4. Phytochemical Constituents

Naphthalene, anthraquinone, and naphthoquinone are the three most notable constituents of the *E. bulbosa* bulb. The compounds isolated from the *E. bulbosa* bulb comprise hongconin, eleutherin, eleutherol, isoeleutherol, isoeleutherine, elecanacin, eleutherinoside A, eleuthoside B, and four polyketides including (R)-4-hydroxyeleutherin, eleuthone, eleutherinol-8-O-β-D-glucoside and isoeleuthoside C (dihydroisoeleutherin-5-O-β-D-gentiobioside) [9,16,17,18]. Other compounds like 9,9′-dihydroxy8,8′-dimethoxy-1,1′-dimethyl-1H, 1H′-[4,4′]bis[naphtha[2, 3-c]funanyl]-3,3′-dione, 6,8-dihydroxy-3,4-dimethoxy1-methyl-anthraquinone-2-carboxylic acid methyl ester and 2-acetyl-3,6,8-trihydroxy-1-methyl anthrax-quinone, were also isolated through bio-guided extraction [19]. Moreover, our recent study also revealed eight bioactive compounds from *E. bulbosa* bulb, i.e., eleutherin, gallic acid, chlorogenic acid, quercetin, kaempferol, rutin, epicatechin gallate, and myricetin [20]. Table 1 shows the examples of the predominant bioactive constituents isolated from the *E. bulbosa* bulb.

## 5. Pharmacological Activities

### 5.1. Chemotherapeutics: Potential Cytotoxic Agent

The studies on the cytotoxic properties of the *E. bulbosa* bulb are minimal and more investigations are required to explore its maximum potential further. Literature studies have demonstrated that the *E. bulbosa* bulb exhibited potent cytotoxic properties on several cancer cells in vitro. For example, it displays a potent cytotoxic effect on a mouse lymphocytic leukemia cell line (L1210) with a half inhibitory concentration (IC_50_) of 9.56 ppm [25]. However, an in-depth study is needed to assess the mechanism within.

Apart from that, two combinations of the *E. bulbosa* bulb extract and doxorubicin displayed a potent cytotoxic effect towards cervical cancer cells (HeLa) synergistically rathter than doxorubicin alone, with 10 ppm and 50 nM, as well as 15 ppm and 50 nM, respectively [26]. The combinative index recorded were 0.67 and 0.70, proposing a good synergistic effect of both compounds on cervical cancer cells. Other combinations exhibited antagonistic effects on cervical cancer cells that may be due to doxorubicin-resistance cells. Similarly, Mutiah et al. [27] investigated the synergistic effect of *E. palmifolia* and *Macrosolen cochinchinensis* on HeLa cancer cells. The results revealed that the combinative treatment offered potent synergistic activity by inducing cell cycle arrest at G_0_-G_1_, G_2_M, and S phases [27]. In addition, the number of apoptotic cells was also increased upon treatment on HeLa cancer cells, indicating that both treatments promote synergistic activity on the cancer cells, which could also be a potential chemotherapeutic agent [27].

A study conducted by Rani [28] revealed that the Soxhlet extraction of hexane and ethyl acetate extracts from the *E. bulbosa* bulb extracts exhibited promising cytotoxic effects on Dalton’s Ascites Lymphoma (DLA) cell line, with half lethal concentration (LC_50_) of 67.97 μg/mL and 41.02 μg/mL, respectively. Meanwhile, Lubis et al. [29] conducted a cytotoxic study on adenocarcinoma colon cancer cells, WiDr, and found that the maceration extraction of *n*-hexane extract of the *E. bulbosa* bulb displayed a selective cytotoxic on the cells with IC_50_ value of 56.15 μg/mL compared to ethyl acetate (70.758 μg/mL) and ethanol (364.103 μg/mL ) extracts, respectively. Similarly, Li et al. [30], who conducted on three different colon cancer cells, HCT116, DLD1, and SW480 cells, using isolated compounds from the *E. palmifolia* bulb, revealed that eleutherinoside C and isoeleutherin compounds presented selective cytotoxic properties on the cancer cells and inhibited the transcription of TCF/ß-catenin in SW480 cancer cells in a dose-dependent manner, compared to the positive control, quercetin.

### 5.2. Endocrine Disorder: Diabetes Mellitus

Diabetes mellitus is a severe endocrine disorder characterized by chronic hyperglycemia, causing multiple vascular complications and cardiac dysfunction [31]. In addition, studies have shown that the increased glucose level or hyperglycemia could lead to various endothelial dysfunctions, e.g., dysregulation of endothelial proliferation, migration, and apoptosis [32]. Therefore, therapeutic approaches should be enhanced to prevent hyperglycemic-induced oxidative stress, to reduce the risk of diabetic complications [33]. Recent studies involving the *E. bulbosa* bulb would be discussed as a functional bioactive agent for diabetic research, in vitro and in vivo.

#### 5.2.1. In Vitro Studies

A study conducted by Chen et al. [21] revealed that the new naphthalene derivatives isolated from the *E. americana* bulb such as eleutherol A, B, and C, eleuthinone B, and C demonstrated a protective effect against human umbilical vein endothelial cells (HUVECs) induced by high glucose level, in vitro. Furthermore, the other three known compounds, i.e., hongconin, Karwinaphthol A, and dihydroisoeleutherin, also showed a similar protective effect compared to the positive control, mannitol. Thus, the findings demonstrated the potential therapeutic effect of the isolated compounds from the *E. bulbosa* bulb against hyperglycemia.

Next, Lahrita et al. [34] reported that the bulb of *E. americana* manifested insulin-induced glucose uptake-enhancing activity at a concentration of 50 μg/mL, including 12 other medicinal plants such as *Andrographis paniculate* (Burm. F.), *Areca catechu* L. fruits, *Caesalpinia sappan* L. wood, *Eurycoma longifolia* Jack root, *Moringa oleifera* Lam. leaves, *Piper betle* L. leaves, *Piper crocatum* leaves, *Piper nigrum* L. fruits, *Syzygium aromaticum* (L.) Merr. and L. M. Perry fruits, *Terminalia bellirica* (Gaertn.) Roxb. fruits, *Tinospora crispa* (L.) Hook. bark, and *Pimpinella anisum* L. seeds compared to rosiglitazone. All the medicinal plants are traditionally utilized in Indonesia to manage diabetic symptoms [34]. The study also indicated their potential efficacy for diabetic treatment by promoting insulin sensitivity.

Similarly, an isolated compound like eleutherinoside A extracted from the *E. americana* methanolic bulb extract proved to be a potent α-glucosidase inhibitor in vitro, with an IC_50_ of 0.5 mM [3]. Eleutherol and eleuthoside B were also successfully isolated but displayed less inhibitory action on α-glucosidase activity (IC_50_ > 1.0 mM). The study suggested that eleutherinoside A was the main inhibitory compound for α-glucosidase activity, even though the inhibition was relatively low compared to a commercially available glucosidase like acarbose. Besides, this study could also benefit in search of novel alternatives for diabetic management, as reported by [3].

#### 5.2.2. In Vivo Studies

Febrinda et al. [35] reported that the intake of *E. palmifolia* aqueous and ethanolic bulb extracts presented an increase in body weight of the alloxan-induced rats compared to the diabetic rats. However, the increment of the body weight in the diabetic-treated group was similar to the non-diabetic untreated group, suggesting that the intake of the extracts did not affect the overall body weight. The blood serum glucose level in diabetic rats revealed a significant increase compared to the non-diabetic untreated group after 28 days. However, the intake of the aqueous and ethanolic bulb extracts significantly improved the blood glucose level in diabetic rats but did not cause any effect in non-diabetic rats, suggesting that the extracts were very selective. The aqueous and ethanol extracts also reduced the total cholesterol (TC) (62.55 ± 11.53 mg/dL, 55.03 ± 15.92 mg/dL) and low-density lipoprotein (LDL) (32.70 ± 7.92 mg/dL, 41.78 ± 10.72 mg/dL) level significantly in diabetic rats rather than glibenclamide-treated diabetic rats (90.45 ± 6.25 mg/dL, 77.33 ± 10.75 mg/dL, respectively). Renal functions were also improved as the serum albumin level was significantly increased with the administration of the aqueous (4.16 ± 0.54 mg/dL) and ethanol (4.69 ± 1.02 mg/dL) extracts in contrast to the untreated diabetic group (2.71 ± 0.33 mg/dL), and no hepatic toxicity was observed. Overall, this study provides an evidence-based medicinal report for the traditional use of *E. bulbosa* bulbs among the local Dayak community to manage diabetes by inhibiting the alpha-glucosidase enzymes that could reduce the postpandrial blood glucose level.

Ahmad et al. [36] reported that the methanolic extract of the *E. palmifolia* bulb using three distinct extraction methods demonstrated a reduction in glucose tolerance level in Swiss albino rats. The results showed a significant reduction using the reflux and maceration methods, with a percentage glucose level of 62.2% and 74.6% at 90 min post-treatment, respectively, in contrast to the positive controls, glibenclamide (55.17% ± 9.55). Meanwhile, Nurcahyawati et al. [37] documented that the extract of the *E. bulbosa* bulb protected the kidney of alloxan-induced Wistar rats. A dose of 400 mg/kg of *E. bulbosa* bulb was sufficient to protect the degeneration of kidney tubule, tubular and globular necrosis, as well as interstitial infiltration compared to the positive control, metformin.

### 5.3. Infectious Disease: Microbial Activity

The infectious disease accounts for approximately a quadrant of overall mortality cases worldwide annually [38]. All infectious pathogens like viruses, bacteria, fungi, protozoa, and worms, share a similar pathologic feature by colonizing the host cells. Therefore, natural products have been extensively studied to overcome resistance and the newly emerging pathogens. For example, the bulb of *E. bulbosa* has been reported as an excellent anti-microbial agent due to its leading bioactive compounds such as naphthalene, anthraquinone, and naphthoquinone.

#### 5.3.1. Anti-Bacterial Properties

Munaeni et al. [39] reported that the bulb of *E. bulbosa* extract demonstrated a significant increase in immunity response and developed resistance towards *Vibrio parahaemolyticus* infection in white shrimp *Litopenaeus vannamei* with a concentration of 12.5 g/kg after 30 days. The immune responses, such as the total hemocyte count, phenoloxidase activity, respiratory burst, and total bacterial count, revealed a significant increase and gene expression activity like prophenoloxidase (proPO), lipopolysaccharide- and β-1,3-glucan-binding protein. The bulb of *E. bulbosa* could also suppress *V. parahaemolyticus* in the intestines, hepato-pancreas, and muscles, thus increasing the survival rate of the white shrimp. In another study conducted by Munaeni et al. [40], the *E. bulbosa* ethanolic bulb extract was shown to significantly inhibit the growth of *Vibrio harveyi* in a dose-dependent manner compared to chloramphenicol. The inhibition diameter increases proportionate to the concentrations, while, phytochemical studies revealed the presents of flavonoid, alkaloid, quinones, and triterpenoids.

Next, Jiang et al. [41] investigated the anti-microbial activity using the active fractions extracted from the *E. bulbosa* bulb against pathogenic bacteria like *Staphylococcus aureus*, *Escherichia coli*, and *Pseudomonas aeruginosa*. The results documented that eleubosa A and B compounds displayed a moderate inhibitory activity against *E. coli* with a minimum inhibitory concentration (MIC) value of 12.5 μg/mL. Meanwhile, a mild inhibitory activity was recorded against *S. aureus* and *P. aeruginosa* with MIC values of 25 μg/mL as compared to the positive control, chlarithromycin. Similarly, a research performed by Mahmudah et al. [42] indicated that the water extract of the *E. palmifolia* bulb inhibited the growth of *E. coli* significantly, with an inhibition diameter of 6 mm at the lowest concentration of 10% and 30 mm at the highest concentration of 100% as compared to the positive control, ceftriaxone (35 mm).

Sirirak et al. [43] evaluated the anti-adhesive and invasion properties of *Campylobacter* spp. on colon cancer cells (Caco-2) using the *E. americana* bulb extract. The results revealed that the preincubation of the bulb extract with the bacterial isolates prevented the adhesive and invasion effects of *Campylobacter* spp. on the Caco-2 cells, with no significant cytotoxic effects at the highest concentration (250 μg/mL) of the bulb extract compared to chloramphenicol. Thus, the study envisaged the importance of the *E. americana* bulb extract as a valuable source of food preservative to control *Campylobacter* contamination. A prior study by Sirirak and Voravuthikunchai [44] also demonstrated the inhibition of 65 *Campylobacter* spp. using Thai medicinal plants such as *Rhodomyrtus tomentosa* (Aiton) Hassk., *Quercus infectoria* G. Oliver, and *E. americana* Merr. The results revealed that both *R. tomentosa* and *Q. infectoria* extracts presented mild anti-microbial activity against *Campylobacter* spp., with the percentage inhibition of 36% and 16%, 46% and 16% against human and chicken sample isolates, respectively. Correspondingly, the *E. americana* ethanolic bulb extract displayed good antibacterial activity against all tested isolates, with an inhibition diameter ranging from 10 to 37 mm. Moreover, the MIC values recorded were 31.25 to 500 μg/mL and 62.50 to 1000 μg/mL for human and chicken isolates of *Campylobacter* spp. in contrast to the positive control, chloramphenicol. Moreover, the extract recorded the minimum bactericidal concentration (MBC) values of 31.25 to 1000 μg/mL and 125 to 1000 μg/mL for human and chicken isolates, respectively, indicating that the results were comparatively as effective as other studies conducted by Tan et al. [45] and Lee et al. [46] The results of the time-kill assay revealed that 2 MIC and 4 MIC doses ultimately reduce the growth of *Campylobacter* strains after 18 h and the isolates from human and chicken samples.

A study conducted by Harlita and Oedjijono [47] demonstrated that the *n*-hexane, ethyl acetate, and 96% ethanol of *E. bulbosa* bulb extracts exhibited a good microbial inhibition against pathogenic bacteria such as methicillin-resistant *Staphylococcus aureus* (MRSA), *Bacillus cereus*, *Shigella* sp., and *Pseudomonas aeruginosa* in the disk-diffusion agar method. The ethyl acetate extract displayed the highest inhibition (10 mg/mL) on the growth of *P. aeruginosa* and *S. aureus* compared to cefadroxil (30 mg/mL). The thin-layer chromatography (TLC) revealed the presence of alkaloid compounds, indicating that the microbial inhibition was caused by the interference between alkaloid compounds and the formation of peptidoglycans components, disrupting the microbial cell wall [47,48]. A preliminary study conducted by Padhi and Panda [49] found that the butanol extract of the *E. bulbosa* bulb displayed a good inhibition zone against *S. aureus* and *Shigella boydii* compared to gentamicin and ciprofloxacin.

The multidrug bacterial resistance strains, such as MRSA, have emerged as the cause of potentially fatal diseases that included necrotizing fasciitis, pneumonia, endocarditis, and acute sepsis in the last decade [50]. An investigation conducted by Ifesan et al. [8] on the mode of anti-staphylococcal inhibition by *E. americana* revealed that the MIC values ranged between 62.5 and 1000 μg/mL, indicating an excellent inhibitory action against *S. aureus* activity. All MRSA isolates in the study were resistant to oxacillin, with MIC values ranging from 80 to 1280 μg/mL. Apart from that, the halo-tolerance assessment was also conducted to determine their growth activity, as several reports discovered that they could survive in extreme salinity [51,52]. The combination of the *E. americana* ethanolic bulb extract (0.5 MIC) and 7.5% sodium chloride (NaCl) displayed a synergistic effect, with a reduced growth rate of *S. aureus*, suggesting that it could be useful for preserved food. Moreover, the observation under transmission electron microscopy (TEM) demonstrated morphological changes within the outer membrane of *S. aureus*, i.e., cell leakage at the concentration of 2 MIC and 4 MIC. Similarly, Ifesan et al. [53] confirmed that the ethanol and acetone extracts of *E. americana* bulb showed good inhibitory activity against *S. aureus* isolated from 106 food samples. In another study, Ifesan et al. [54] revealed that the partially purified fraction of the *E. americana* bulb extract inhibited the MRSA food isolates, with MIC and MBC values (Ea6.3) of 125 to 500 μg/mL and 250 to 1000 μg/mL, respectively. Another active fraction, Ea9.0, yielded MIC and MBC values of 125 to 250 μg/mL and 500 to 1000 μg/mL, respectively. The Ea6.3 extract managed to inhibit the reference strain (ATCC 27664) at a concentration of 4 MIC (1000 μg/mL) and 2 MIC within 20 h and 24 h. For the MRSA isolates, the extract suppressed the growth of NPRC 421 and NPRC 461 after 24 h. In the meantime, the Ea9 extract displayed a lethal effect on enterotoxin-producing reference strain at a concentration of 4 MIC after 24 h. All extracts were compared with vancomycin on the MRSA isolates.

In one study conducted by Limsuwan and Voravuthikunchai [55], three medicinal plants from Thailand, i.e., *Boesenbergia pandurata* (Roxb.) Schltr., *E. americana* Merr. and *R. tomentosa* (Aiton) Hassk, were tested on the biofilm formation of *Streptococcus pyogenes*. The results demonstrated that *R. tomentosa* extract showed potent inhibition against the growth of *S. pyrogene*, while, *E. americana* only displayed a partial inhibitory activity. The evaluation of biofilm formation under the microscope revealed that *S. pyrogene* (NPRC 109) was suppressed when treated with the sub-inhibitory concentration of the extracts. Moreover, anti-quorum sensing was also exhibited by the extracts by the loss of the violet pigment violaceine in *Chromobacterium violaceum*. Meanwhile, the results of Maftuch et al. [56] demonstrated that the *E. palmifolia* ethanolic bulb extract displayed anti-microbial activity against *Aeromonas hydrophila*-infected *Cyprinus carpio* through a histopathology study of gills, kidney, liver, and muscle. The treatment also significantly demonstrated wound healing properties towards the infected tissues in a dose-dependent manner. On top of that, Panda et al. [57] reported that several extracts exhibited strong MIC values (<1 mg/mL) against a few Gram-positive and Gram-negative bacteria, including the *E. bulbosa* bulb. The bulb extract documented the lowest MIC values ranging from 22 to 125 μg/mL on the growth of *Shigella flexneri*, *Vibrio cholerae*, *B. cereus*, and *S. aureus*. However, the bulb extract only showed moderate inhibitory activity against the bacteria in a disc diffusion method with inhibition zones of 15 to 20 mm.

#### 5.3.2. Anti-Fungal Properties

Fungal infections are a global threat to human health, with estimated cases of more than a billion skin and mucosal infections, allergies, as well as death each year [58]. Fungal infections could trigger a complex set of systemic diseases resulting from fungal virulence factors that could eventually lead to tissue damage and inflammation. Natural resources offer several bioactive compounds, transcending in the anti-microbial fields, with the first commencement of antibiotic development, penicillin [59].

Masfria and Tampubolon [60] revealed that the *E. palmifolia n*-hexane bulb extract demonstrated strong inhibitory activities against *Candida albicans* and *Trichophyton mentagrophytes* at a concentration of 200 mg/mL and 20 mg/mL. The microbial inhibition displayed a clear diameter zone of 19.48 mm and 21.02 mm for *C. albicans* and *T. mentagrophytes*, respectively, suggesting a superior anti-microbial activity. The extract induced a change in the membrane permeability against pathogens, causing cell death and molding [61,62]. Besides, the inhibition was also caused by the presence of terpenoids extracted using a non-polar solvent with lipophilic components that could damage the cell membrane of the pathogens [63].

The essential oil extracted from the *E. bulbosa* bulb was also a good inhibitor against *Malassezia furfur*, a fungus infection on the stratum corneum of the skin, which could form pityriasis versicolor [64]. Hayati et al. [64] reported that the highest inhibition yielded a diameter of 9.25 mm at a concentration of 50%, while the lowest inhibition was only 1.75 mm at a concentration of 3.125%, which was still relatively low compared to ketoconazole, the positive control with an inhibitory diameter of 20 mm. However, ketoconazole usage against *M. furfur* causes multiple side effects such as skin burning, irritation, and itching, suggesting that an alternative treatment like the *E. bulbosa* bulb should be further investigated as a potential anti-fungal infection.

In a study carried out by Kusuma et al. [1], they reported that eleutherin isolated from *E. bulbosa* bulb significantly inhibited *T. mentagrophytes* in the agar diffusion assay, and it was believed that it might display fewer adverse effects in topical application as compared to miconazole. Correspondingly, Alves et al. [23] conducted a study on the growth of the *Clasdosporium sphaerospermum*, a pathogenic fungus of plants and marine species, using the *E. bulbosa* bulb extract. The *E. bulbosa* bulb extract demonstrated potent inhibitory activity in a bioautography assay. Moreover, the experiment was further continued for fractionation of the compounds, and four bioactive compounds, i.e., eleutherinone, eleutherin, isoeleutherin, and eleutherol, were isolated. The compounds demonstrated potent anti-fungal activities at 100 μg/spot, except for eleutherinone, which was inactive.

#### 5.3.3. Anti-Viral Properties

In a study by Hara et al. [16], it was found that isoeleutherine and isoeleutherol isolated from the bulb of *E. americana* demonstrated potential anti-viral agent against HIV replication with IC_50_ values of 8.5 μg/mL and 100 μg/mL, respectively. However, more studies should be conducted to validate the effectiveness of the bulb extract as anti-viral agents.

#### 5.3.4. Anti-Malarial Properties

Vale et al. [65] demonstrated that the IC_50_ value of ethyl acetate extract (10.22 ± 2.32 μg/mL), eleutherin (10.45 ± 3.13 μg/mL), isoeleutherin (8.70 ± 2.45 μg/mL), and S2 fraction (eleutherin + isoeleutherin, 3.67 ± 1.01 μg/mL) extracted from the bulb of *E. plicata* showed good anti-plasmodial activity compared to the positive control, chloroquine (0.023 ± 0.009 μg/mL). The results indicated that the naphthaquinone compounds eleutherin and isoeleutherin were responsible for the anti-plasmodial activity. In silico analysis through molecular docking suggested that the isoeleutherin and eleutherin could be potential anti-malarial drugs, causing mitochondrial damage on the parasite.

### 5.4. Anti-Inflammatory Activities

Felix Hoffman was the first German chemist who created the world’s most usable therapeutic agent, aspirin, back in sesquicentennial [66]. It acts as an inhibitor of cyclooxygenase (COX) enzymes like COX-1 and COX2 by blocking the inflammatory mediators, i.e., prostaglandins and thromboxane [66]. Many inflammatory mediators occur during inflammatory responses upon harmful stimuli that trigger the immune system’s alarm on the injured tissues. Pro-inflammatory cytokines like tumor necrosis factor (TNF)-α, interleukin (IL)-1β, and vascular endothelial growth factor (VEGF) are the primary drivers during the dynamic inflammatory responses [66]. However, current anti-inflammatory drugs for chronic diseases such as cancer, rheumatoid arthritis, diabetes, and autoimmune disorders instigate concomitant side effects on the glucocorticoid hormone cortisol in the long run. Thus, an alternative approach that could promote effective, economic, and modulatory drugs is fundamental. Natural resources like plants could be a promising avenue in drug discovery due to their relative abundance and economic cost.

#### 5.4.1. Rheumatoid Arthritis

In one study conducted by Hanh et al. [67], the intake of *E. bulbosa* ethanolic bulb extract reduced the incidence of arthritis in collagen antibody-induced arthritis mice, in vivo. The inflammation, swelling, and redness was significantly reduced at a 1000 mg/kg extract concentration after 10 days of treatment compared to dexamethasone. Interestingly, the intake of 500 mg/kg extract demonstrated a slight decrease in the arthritis score, which gradually improved at the end of the experiment. No significant difference was recorded between the given extracts of 500 and 1000 mg/kg after 10 days. The histopathology reviews documented that the edema was reduced, while the infiltration of inflammatory cells in the joints was observed at 1000 mg/kg. Moreover, the pro-inflammatory cytokines like TNF-α and IL-6 were significantly suppressed after administering the 1000 mg/kg extract. Above all, the extract displayed no sign of toxicity at the highest dose of 5000 mg/kg, suggesting that it was not toxic according to the World Health Organization (WHO) and Organization for Economic Cooperation and Development (OECD). In an in vitro study on lipopolysaccharide (LPS)-induced RAW 264.7 macrophages, the extract presented a potent inhibitory of nitric oxide production (NO) with an IC_50_ value of 27.30 μg/mL. Overall, the study suggested that the *E. bulbosa* bulb could be a potential anti-inflammatory agent as the use of tocilizumab and anti-TNF-α monoclonal antibodies in rheumatoid arthritis may escalate liver transaminase and lipid levels.

#### 5.4.2. Erythrocyte Membrane Stabilization

In a research report performed by Paramita and Nuryanto [68], the ethanolic bulb of *E. bulbosa* demonstrated a potential anti-inflammatory activity on the erythrocyte membrane stabilization compared to the positive control, indomethacin. The half-maximal effective concentration (EC_50_) recorded for the *E. bulbosa* bulb extract was 52.87 μg/mL, while the EC_50_ value for indomethacin was 26.39 μg/mL.

#### 5.4.3. Lipopolysaccharide-Induced (LPO) Nitric Oxide Production

Song et al. [69] disclosed that isoeleutherin isolated from the *E. americana* bulb inhibited the activity of LPS-stimulated RAW 264.7 macrophage cells NO production in a dose-dependent manner, with an IC_50_ value of 7.4 μM. Furthermore, cell viability recorded more than 95% survival, indicating that cytotoxic properties did not mediate the inhibition of NO production. Meanwhile, mRNA expression revealed the inhibition of inducible nitric oxide synthase (iNOS) and pro-inflammatory cytokines upon treatment with isoeleutherin in a concentration-dependent manner. In addition, the treatment of isoeleutherin markedly inhibited the LPS-induced transcriptional activity of NF-κB in a dose-dependent manner, indicating that the suppression of iNOS by isoeleutherin was associated with the regulation of the NF-κB transcription factor.

In another study by Han et al. [70], it was documented that the isolated compounds from the *E. americana* bulb such as isoeleutherin, eleutherin, hongconin, dihydroeleutherinol, and eleutherinol displayed a potent inhibitory activity on LPS-activated mouse macrophage RAW 264.7 cells, with IC_50_ values of 7.7, 11.4, 19.8, 21.7, and 34.4 μM, respectively. Compounds like isoeleutherin and eleutherin demonstrated a stronger inhibitory activity than the positive control, *N*-methylarginine (_L_-NMMA). The authors also suggested that naphthoquinone and naphthalene groups with pyran or lactone rings were excellent for inhibiting NO production.

#### 5.4.4. Activation of CD4+ T Helper (Th) Cells

In a study conducted by Hong et al. [71], it was reported that the treatment of 10 μM isoeleutherin isolated from the *E. americana* bulb did not affect the IL-2 production. However, it displayed a significant increase in the interferon-gamma (IFNγ) production at 24 and 48 h post-T-cell receptor (TCR) stimulation. The intracellular cytokine staining results also demonstrated a significant increment of IFNγ cell populations by isoeleutherin, suggesting that it could be an IFNγ precise stimulator. Moreover, the production of IFNγ is regulated by the Th-1 specific transcription factor called T-bet, which helps differentiate Th-1 cells. The results of quantitative real-time polymerase chain reaction (qPCR) results revealed that the treatment of isoeleutherin enhanced both IFNγ and T-bet expressions in a concentration-dependent manner. Moreover, isoeleutherin could modulate the IFNγ production in a T-bet dependent manner, especially in wild-type Th cells compared to T-bet deficient cells. On the contrary, eleutherinol was found to inhibit IFNγ and IL-2 production in a semi-quantitative RT-PCR significantly. Therefore, it is concluded that isoeleutherin is a stimulant of the Th-1 immune response, while eleutherinol inhibited cytokine production during T cell activation.

### 5.5. Dermatological Conditions

#### 5.5.1. Wound Healing Properties

Skin is the body’s largest organ, covering the outer layer of an organism and ectodermal tissues, acting as a safeguard for internal organs, muscles, ligaments, and bones [72]. Injuries caused by external stimuli may form a wound that could compromise the structure and function of the skin. For this reason, wound healing plays functional properties as complex and dynamic feedback to repair the disrupted anatomical function of skin [72]. It requires a gradual progression of events like inflammation, granulation, contraction, collagen formation, epithelization, and cicatrization to restore the disrupted anatomical site [73]. Any disturbance during the process may affect tissue rejuvenation and cause chronic tissue damage, infections, or scarring. The wound healing process could be assisted with natural products due to their synergistic bioactive effects of flavonoids, phenolic, tannins, alkaloids, and terpenoids. In addition, phytochemicals have been scientifically proven to possess wound healing activity through modulation of essential phases within the process, and good topical bioavailability by the epidermis layer.

An investigation by Rezandaru et al. [74] reported that the topical application of *E. palmifolia* gel extract demonstrated a gradual wound healing activity on alveolar osteitis-induced Sprague–Dawley mice. This was observed in a histopathology test where the infected socket was already packed with granulated tissues like fibroblast and collagen on day 3. In addition, the trabecular teeth bone was also formed at the apical edge, reaching the center of the socket. The progression was further observed as the trabecular bone was almost developed and filled the socket on day 5. In the meantime, fibrosis and osteogenesis were displayed with a trabecular bone covering nearly the whole inflamed socket at day 10. Moreover, the percentage of fibroblast, collagen density, and osteogenesis rate gradually increased from day 3 to 10, according to the Gibson–Corley scoring system. Interestingly, the study displayed that the effectiveness of the gel extract was significantly similar to the positive control, iodoform paste.

An in vivo cutaneous wound healing study conducted by Upadhyay et al. [75] on Wistar rats revealed that the high dose of *Eleutherine indica* methanolic bulb extract was safe for topical application, with no symptoms of irritation and inflammation on the rats. The granulated connective tissues were noticeable after three days post-injury, especially in the methanol extract and gentamicin sulfate-treated group. The treatment with the methanolic bulb extract was presented with a wound contraction of 50% to 87%, while the gentamicin sulfate-treated group exhibited 52% to 92% post-operative on day 20. The hydroxyproline level was also higher when using the bulb extract and was statistically insignificant to the positive control, proposing that they have similar wound healing effects on the injured rats. The histopathological assessment presented a well-regulated wound healing activity in the treated groups (extract and positive control) compared to the non-treated groups and the vehicle control. Post-operative observation on day 15 revealed a more precise outcome in which treatment with the bulb extract and gentamicin sulfate increased the collagen layering and rapid keratinization with intraepithelial cornification. Meanwhile, Western blot analysis revealed an increase in granulation proteins like COL3A1, bFGF, Smad-2, and Smad-4 responsible for connective tissue assembles. The increment of the protein levels was caused by the *E. indicata* methanolic bulb extract, confirming the claim for its traditional use as a wound-healing agent.

#### 5.5.2. Anti-Melanogenesis Activity

Fair skin is a paradigm of beauty in Asian cultures [76]. Therefore, the investment in skin whitening products in the Asian market escalated yearly, especially in countries like China, Japan, and India [77]. The skin color depends on the genetic background and a few external factors like sunlight exposure and environmental pollution. Melanin, a dark-colored pigment, is synthesized by the melanocytes localized in the epidermis layer and responsible for skin color, hair, eyes, and skin homeostasis. The melanin is carried in melanosomes towards the adjacent keratinocytes and allows melanin distribution throughout the epidermis layer of the skin [78]. The most prominent role of melanin is to protect from the harmful ultraviolet (UV) rays and environmental risks of skin damage. Excessive melanin production may not only affect appearances but could also result in several aesthetic problems such as melasma, ephelides or freckles, and post-inflammatory hyperpigmentation (PIH) [77]. Commercially available skin whitening agents, for instance, hydroquinone, mercuric chloride, and corticosteroids showed effective melanocyte inhibition. However, they demonstrated unfavorable side effects that involve dermatitis, irritation, and a prickling sensation [79]. As a result, recent research highlights the benefits of utilizing natural products as a skin-whitening agent to suppress tyrosinase enzyme activity in melanogenesis, subsequently promoting a less cytotoxic effect for human use [80].

A study carried out by Kusuma et al. [1] revealed that the isolated compound from *E. americana*, eleutherin, managed to suppress the formation of melanin in B16 melanoma cells with 87% inhibition at concentrations of 25 and 50 μg/mL compared to arbutin with 53% inhibition at 100 μg/mL. At a concentration of 5 μg/mL, eleutherin inhibited 35% of melanin formation, suggesting that the isolated compound demonstrated potential anti-melanogenesis properties and were safe for further study. In another study performed by Arung et al. [81], eight medicinal plants demonstrated an anti-melanogenesis effect on B16 melanoma cells in contrast to the positive control, kojic acid. These medicinal plants include *E. palmifolia* bulb, *Willughbeia coriacea* bark, *Glochidion philippcum* root, *Eusideroxylon zwageri* seed, *Dendrophthoe petandra* root, *Lansium domesticum* bark, *Passiflora foetida* stem and fruit, and *Solanum torvum* root. The percentage of anti-melanin formation recorded for *E. palmifolia* bulb was 57.3% at the 50 μg/mL concentration. Meanwhile, the percentage of viable cells was up to 83%, suggesting that the extract was not toxic to the cells. The findings also indicated that the inhibition of the melanin activity by *E. palmifolia* bulb extract was caused by the high 1,1-diphenyl-2-picrylhydrazyl (DPPH) radical scavenging activity. Next, Biworo et al. [82] reported that *E. palmifolia* ethanolic bulb extract displayed a low melanin index and hydrogen peroxide (H_2_O_2_) level in UV-induced rats after 24 h of UV exposure. The bulb extract also demonstrated increased erythema and tyrosinase index due to the treatment, suggesting that the *E. palmifolia* bulb may be a potential anti-melanogenesis therapeutic agent.

### 5.6. Antioxidant Activity

External factors like cigarette smoking, alcohol, pollution, and radiation may enhance the rapid production of reactive oxygen (ROS) and reactive nitrogen species (RNS) that could disrupt the homeostatic balance of our body, resulting in oxidative stress [83]. On the other hand, exogenous sources like diet-derived antioxidants are potent scavengers of free radicals that could ameliorate the oxidative damage from chronic and degenerative diseases.

Munaeni et al. [84] reported that the bulb of *E. bulbosa* demonstrated a potent antioxidant activity with an IC_50_ value of 1.48 µg/mL compared to ascorbic acid in DPPH assay. The strong antioxidant activity stimulated the growth of probiotic bacteria like *Pseudoalteromonas piscicida* 1Ub and *Bacillus* sp. NP5, suggesting that the extract of *E. bulbosa* bulb could be a potential prebiotic and antioxidant agent. Our recent study documented that the bulb of *E. bulbosa* displayed strong antioxidant activities in DPPH and ABTS assays under optimized extraction conditions at 75.2% and 74.9% compared to Trolox [20]. The high performance liquid chromatography (HPLC) analysis revealed several bioactive compounds like phenolic and flavonoids, synergistically resulting in a high antioxidant activity. Moreover, it was demonstrated that the antioxidant activity of the *E. bulbosa* bulb could improve the quality of the sperm in mice. Jayanti et al. [85] demonstrated that the bulb extract of *E. bulbosa* significantly increased the sperm concentration in lead acetate-induced mice. The antioxidant effect of *E. bulbosa* was also observed in UV-induced skin oxidative damage in rats [80]. The findings of the study revealed that the bulb of *E. palmifolia* increased the level of superoxide dismutase (SOD), while lowering the production of malondialdehyde (MDA) and advanced oxidation protein products (AOPP) in the treated group.

Shi et al. [86] conducted a study on antioxidant activity from *E. bulbosa* bulb and discovered that the bulb displayed high peroxyl radical scavenging capacity in HepG2 cells. The fluorescent intensity emitted by the DCFH probe also increased in a dose-dependent manner, suggesting that the bulb extract has high phenols and flavonoids that contribute towards its potent antioxidant activity of *E. bulbosa* bulb. In a study carried out by Morabandza et al. [87], the ethanolic bulb extract of *E. bulbosa* displayed higher total phenolic and flavonoid content with 27.12 mg GAE/g DW and 17.97 mg RE/g DW, respectively, compared to aqueous extract. The high content of polyphenols resulted in high antioxidant activity (IC_50_) with 0.595 mg/mL, while the aqueous extract with 1.251 mg/mL. A significant fungal inhibition by *E. bulbosa* ethanolic bulb extract was also observed compared to fluconazole. Agustin et al. [88] reported that 70% methanol displayed the most effective solvent for extracting polyphenols from *E. bulbosa* bulb, with a phenol and flavonoid content of 20 mg GAE/g DW and 15.03 mg QE/g DW compared to gallic acid and quercetin, respectively. Besides, DPPH radical scavenging activity revealed an IC_50_ value of 39.06 µg/mL.

### 5.7. Effect on the Reproductive System Disorders

In a survey conducted by Lans [11], it was reported that *E. bulbosa* extract was used in treating menstrual pain and certain female complaints. However, further pharmacological studies should be investigated as the information to support the study is still scarce [11]. Weniger et al. [10] reported that the bulb of *E. bulbosa* displayed anti-fertility potential in the mouse. Bahtiar and Dewi [89] documented that the synergistic effect of *E. bulbosa* bulb and cowpea extract significantly elevated the calcium level and weight of the bone in ovariectomized rats. The combination of the extracts could also be able to alleviate the level of fat in the bone marrow of the rats, suggesting that a potential application could be further extrapolated in osteoporosis management in post-menopausal women. Similarly, in another study conducted by Bahtiar and Annisa [90], the bulb of *E. bulbosa* significantly increased the calcium level, weight, and bone length in hypoestrogenic rats compared to the tamoxifen-induced group. Other studies also indicated that the *E.bulbosa* bulb could reduce lipids in ovariectomized rats [91].

## 6. The Potential Use of *E. bulbosa* Bulb in Various Applications

### 6.1. Food Industry

#### 6.1.1. Food Additives

Due to its potential biological activities, recent studies presented that the bulb of *E. bulbosa* has been widely used in various applications, e.g., in the food industry. Phoem et al. [92] showed that encapsulation of *E. americana* oligosaccharide extract facilitated the survival of probiotics, *Lactobacillus plantarum* in yogurt. The encapsulation of *E. americana* oligosaccharide extract displayed the highest cell viability as compared to free cells at weeks 2 and 4 upon sequential exposure to stimulated gastric and intestinal juices. The yogurt prepared with encapsulation also revealed less acidification, with lactic acid remained constant for 4 weeks at 4 °C in contrast to the free cells. Moreover, it was shown that the encapsulation method enhanced the anti-microbial activity against enteropathogenic bacteria such as *Clostridium perfringens, S. aureus*, *E. coli*, and *Salmonella typhimurium*, suggesting that the encapsulation of *L. plantarum* and *E. americana* oligosaccharide extract could be a functional food additive.

In another study, Phoem et al. [93] revealed that the microencapsulation of *Bifidobacterium longum* with *E. americana* oligosaccharide extract displayed better survival upon exposure to simulated gastric and intestinal juices for the preparation of alginate complex. Through the extrusion technique, microencapsulation demonstrated better resistance under refrigerated storage and heat treatment compared to free cells after two and four weeks. It was suggested that this study could benefit manufacturing technology for functional foods such as pasteurization due to its ability to withstand heat treatment. Besides that, the microencapsulation of *B. longum* and *E. americana* also presented similar results in fresh milk tofu and pineapple juices [94]. The level of acidification was very low in encapsulated pineapple juice than in free cells. Overall, the bulb of *E. americana* shows great potential as an encapsulating agent for functional foods. In another study led by Phoem and Voravuthikunchai [95], it was revealed that the bulb extract of *E. americana* and oligosaccharide extract demonstrated prebiotic potential on the growth of infant intestinal microbiota and promoted the production of short fatty acids. The findings proposed that the bulb extract of *E. americana* could be supplemented as a functional food due to its prebiotic potential.

Damayanti et al. [96] reported that the addition of *E. americana* bulb extract could improve the antioxidant activity of fermented soybeans (locally known as “nuget tempe”). The results revealed that the mixture of 15% of the bulb extract and the fermented soybeans enhanced the antioxidant content, suggesting that the bulb of *E. americana* improved the scavenging free radical capacity. Ifesan et al. [97] reported that the bulb extract of *E. americana* displayed a promising application in homemade salad dressing. The study indicated that the bulb extract demonstrated good anti-staphylococcal activity and excellent stability in response to heat and pH adjustments. The bulb extract also exhibited additive properties based on the sensory test conducted, while reducing the lipid oxidation in the salad dressing. Thus, it was shown that the bulb extract of *E. americana* could be a potential food additive, especially in homemade salad dressing that could complement vinegar and enhance the overall quality of the food.

The potential food additive of *E. americana* bulb extract was observed in broth and cooked pork against the growth of *S. aureus* [98]. The findings presented that the extract could delay the production of enterotoxins and inhibited the staphylococcal enzymes such as protease and lipase. In another study led by Ifesan et al. [99], the results demonstrated the potential food additive properties from the crude extract of *E. americana* bulb in cooked pork. The potent antioxidant activity of the bulb extract suppressed the lipid oxidation in a dose-dependent manner and inhibited the growth of *S. aureus* after nine days compared to the control. The treatment with the bulb extract also enhanced the redness of the meat, proposing that the bulb of *E. americana* could be a good color enhancer and natural food additive.

#### 6.1.2. Healthy Snacks

Apart from food additives, interest in the *E. bulbosa bulb* for potential snacks was also investigated. In a study performed by Ani et al. [100], stick onions were made from the bulb with a combination of rice flour mixtures. The results showed that the respondents preferred the 1:2 combination (50 g bulb in 100 g rice flour) due to its savory and crispy texture. It provides beneficial information on the bulb as a potential healthy snack; however, extensive studies should be conducted to produce more flavors for the consumer’s choice.

### 6.2. Feed Additives

The bulb of *E. bulbosa* also demonstrated potential uses in animal nutrition. In a study conducted by Ooi et al. [101], the bulb of *E. bulbosa* showed potential application as a feed additive in laying hens. The results indicated that the diet supplemented with 1% of the bulb extract demonstrated a significant reduction of fecal pH and *Enterobacteriaceae* counts, while enhancing the fecal lactic acid bacteria compared to the control treatment. In addition, other medicinal herbs such as turmeric and Vietnamese coriander leaf also possess similar properties, suggesting that these herbs could enhance better performance in laying hens.

Correspondingly, Hardi and Handayani [102] demonstrated that diet supplemented with the bulb extract of *E. bulbosa* enhanced the performance in striped catfishes, *Pangasianodon hypophthalmus*. Furthermore, the study revealed that the supplementation of the *E. bulbosa* bulb extract higher than 30 g/kg could improve the growth rate, amylase, leukocyte, and phagocytosis level in the fishes. Therefore, it was proposed that the bulb extract could be a potential feed additive in catfish nutrition and more studies should be investigated to understand the overall mechanism.

## 7. Conclusions

This review unfolds the botanical description of *E. bulbosa* bulb, phytochemistry, pharmacological activities, as well as its potential application in industry, using multiple scientific names used in different countries in order to obtain the relevant information. Studies on the pharmacological activities from the bulb of *E. bulbosa* revealed potential cytotoxicity, anti-diabetic, anti-microbial, anti-inflammatory, anti-melanogenesis, antioxidant, as well as the treatment of several reproductive system disorders, in vitro and in vivo. It could be proposed that the exceptional source of phytochemicals like naphthalene, anthraquinone, and naphthoquinone contributes to its potent pharmacological activities. Nevertheless, the current knowledge on the mechanism of action is still scarce, calling for more quality studies involving animals and humans. Besides that, the bulb of *E. bulbosa* also demonstrated significant potential applications in the food industry and animal nutrition. However, a detailed exploration should also be conducted to further an in-depth perspective of its maximum potential.

## Figures and Tables

**Table 1:** Chemical constituents isolated from *Eleutherine bulbosa* (Mill.) Urb. bulb.

Chemical Constituents	Phytochemical Family	IUPAC Names *	Chemical Structures	References
Hongconin	Naphthalene	(1*R*,3*R*)-5,10-dihydroxy-9-methoxy-1,3-dimethyl-1*H*-benzo[g]isochromen-4-one	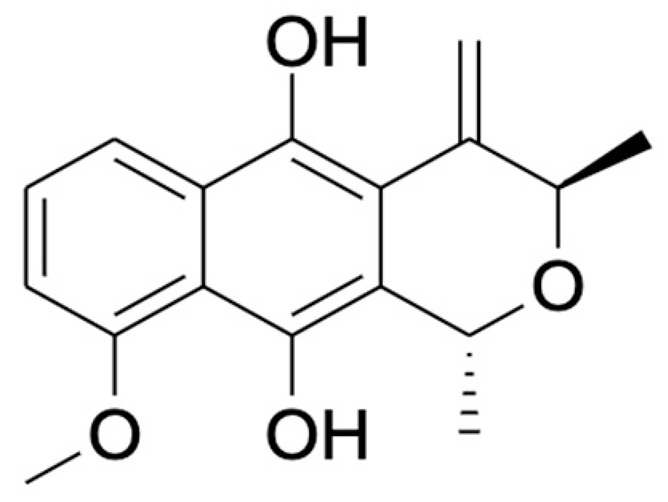	[9]
Eleutherin	Naphthoquinone	(1*R*, 3*S*)-9-methoxy-1,3-dimethyl-3,4-dihydro-1*H*-benzo[g]isochromene-5,10-dione	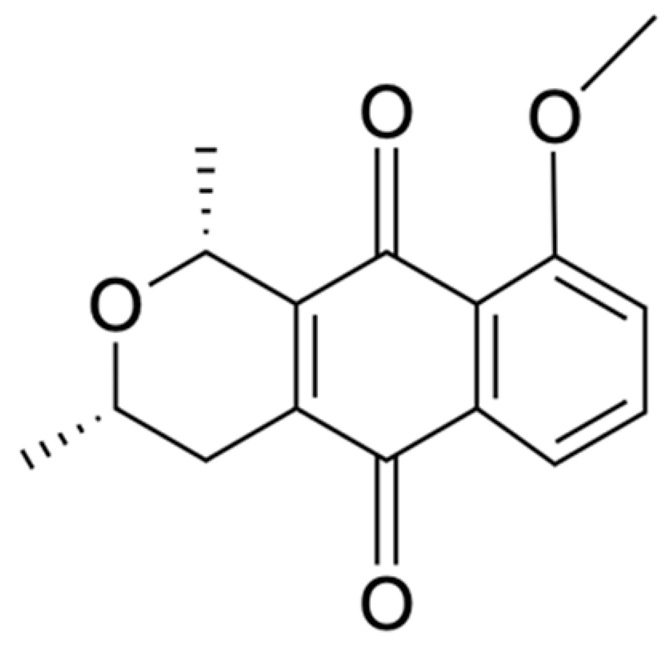	[17]
Eleutherol	Naphthalene	(3*R*)-4-hydroxy-5-methoxy-3-methyl-3*H*-benzo[f][2]benzofuran-1-one	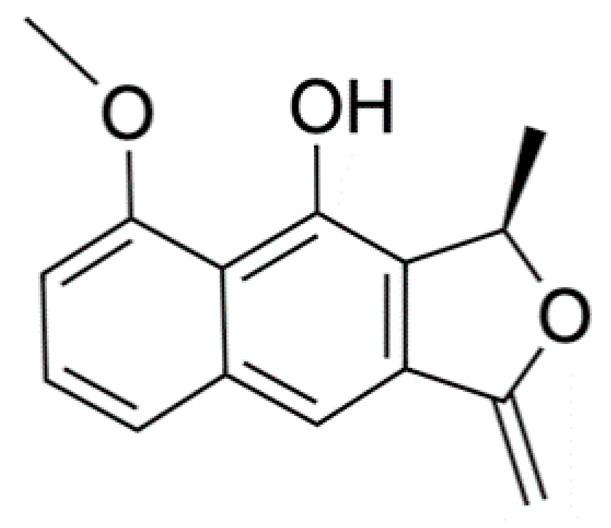	[17]
Eleutherol A	Naphthoquinone	-	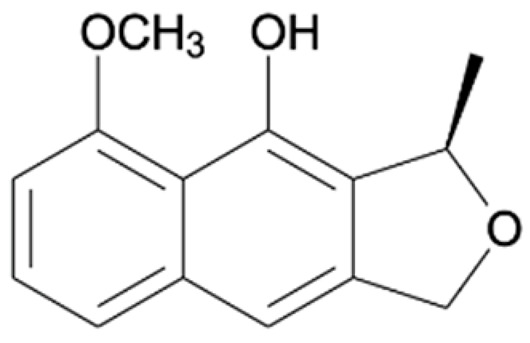	[21]
Eleutherol B	Naphthoquinone	-	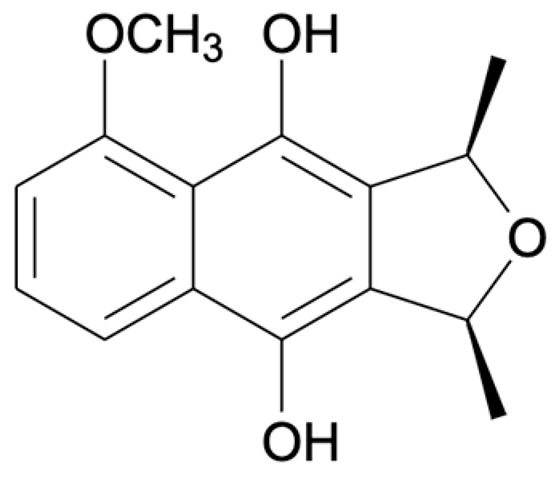	[21]
Eleutherol C	Naphthoquinone	-	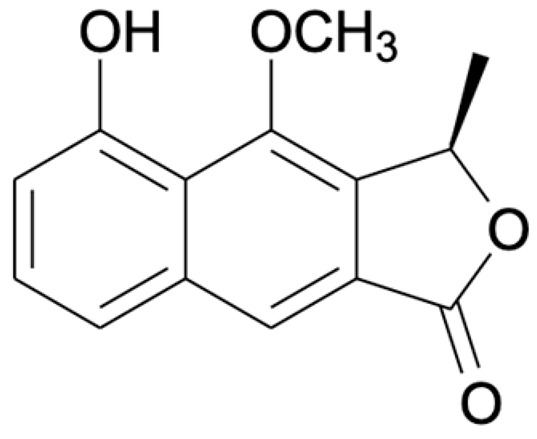	[21]
Eleuthinones B	Naphthoquinone	-	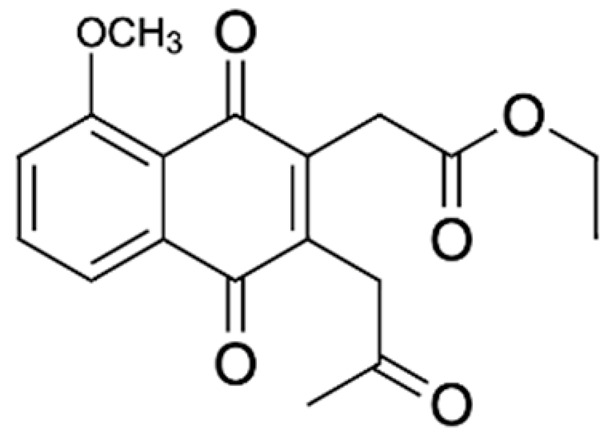	[21]
Eleuthinones C	Naphthoquinone	-	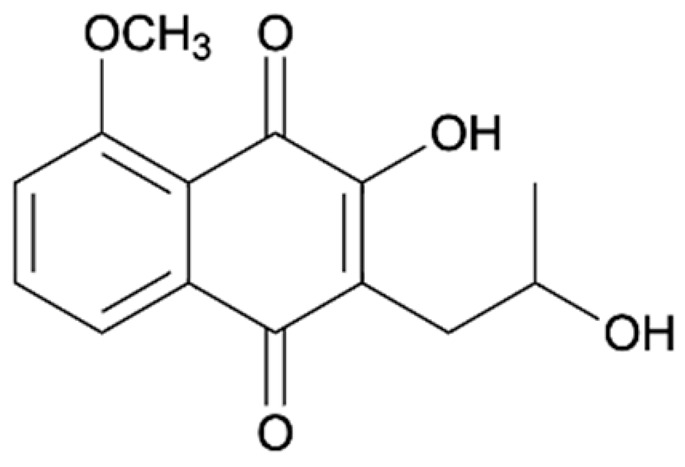	[21]
Eleutherinoside A	Naphthalene	10-hydroxy-2,5-dimethyl-8-[(2*S*,3*R*,4*S*,5*S*,6*R*)-3,4,5-trihydroxy-6-(hydroxymethyl)oxan-2-yl]oxybenzo[h]chromen-4-one	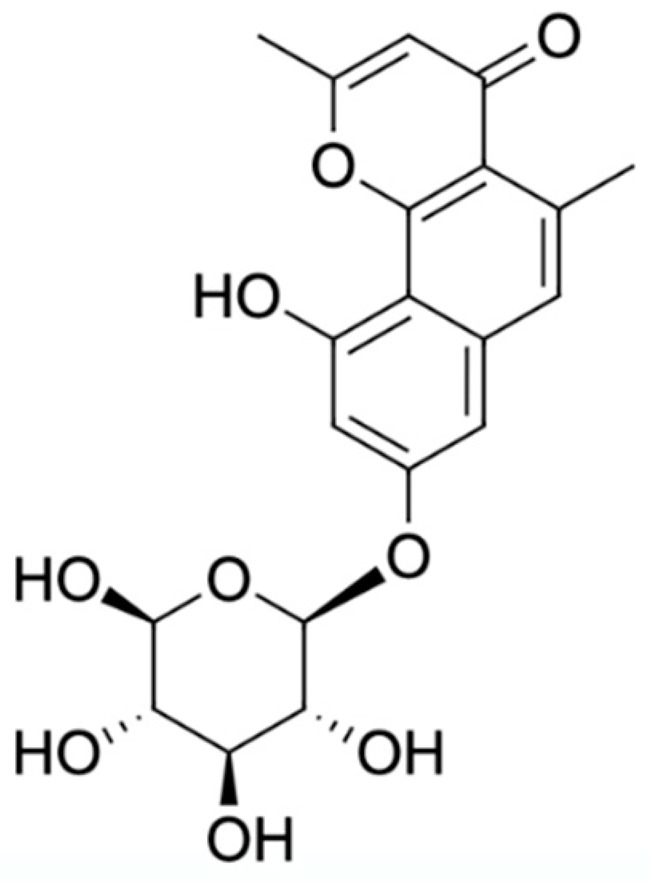	[22]
Eleuthoside B	Naphthalene	(3*R*)-5-methoxy-3-methyl-4 [(2*S*,3*R*,4*S*,5*S*,6*R*)-3,4,5-trihydroxy-6-[[(2*R*,3*R*,4*S*,5*S*,6*R*)-3,4,5-trihydroxy-6-(hydroxymethyl)oxan-2-yl]oxymethyl]oxan-2-yl]oxy-3*H*-benzo[f][2]benzofuran-1-one	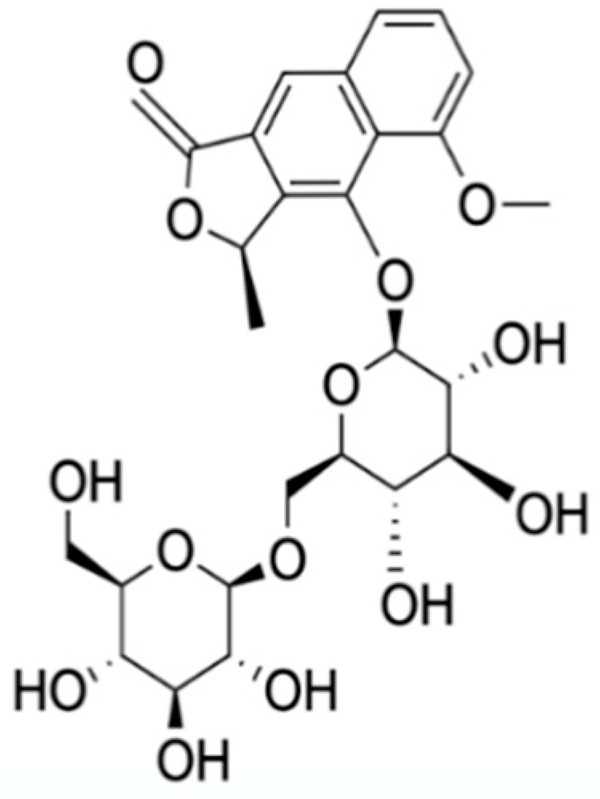	[9,16]
Elecanacin	Naphthoquinone	6-Methoxy-2-methyl-1,2,4,4a-tetrahydro-3aH-naphtho[2′,3′:2,3]cyclobuta[1,2-b]furan-5,10-dione	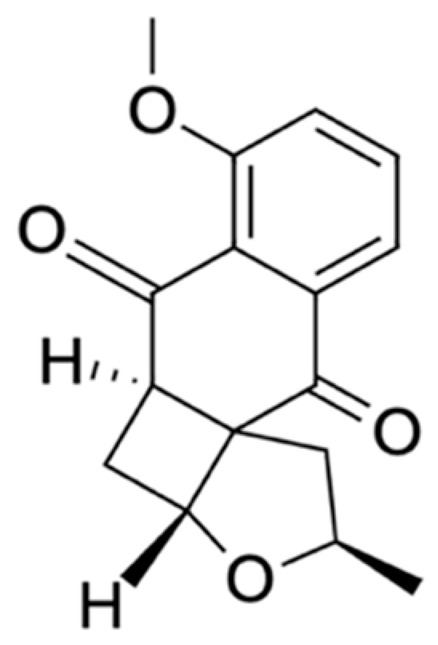	[9,16]
Eleutherinone	Naphthoquinone	8-methoxy-1-methyl-1,3-dihydro-naphtho(2,3-c)furan-4,9-dione	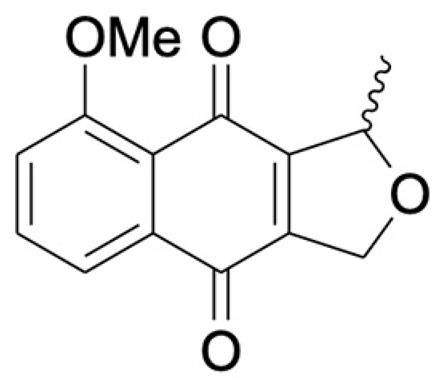	[23]
Isoeleutherin	Naphthoquinone	(1*R*,3*R*)-9-methoxy-1,3-dimethyl-3,4-dihydro-1H-benzo[g]isochromene-5,10-dione	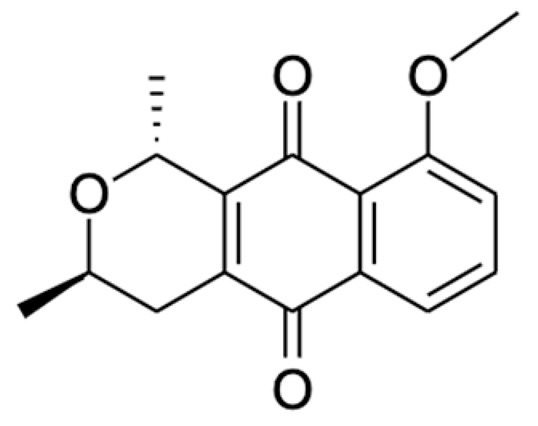	[23]
Eleucanainones A	Naphthoquinone	-	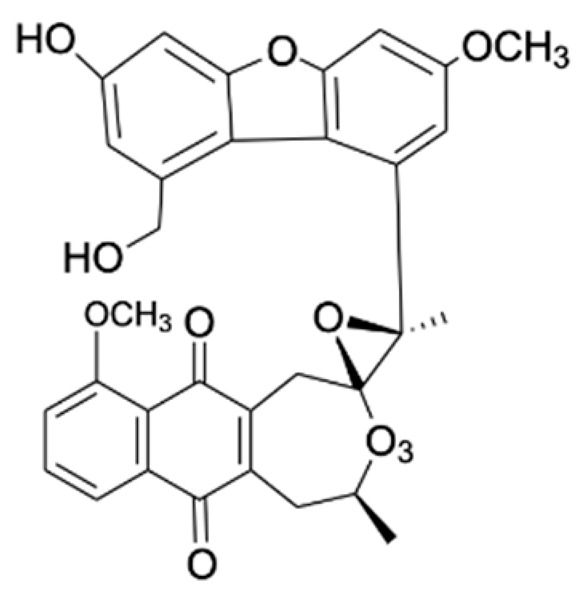	[24]
Eleucanainones B	Naphthoquinone	-	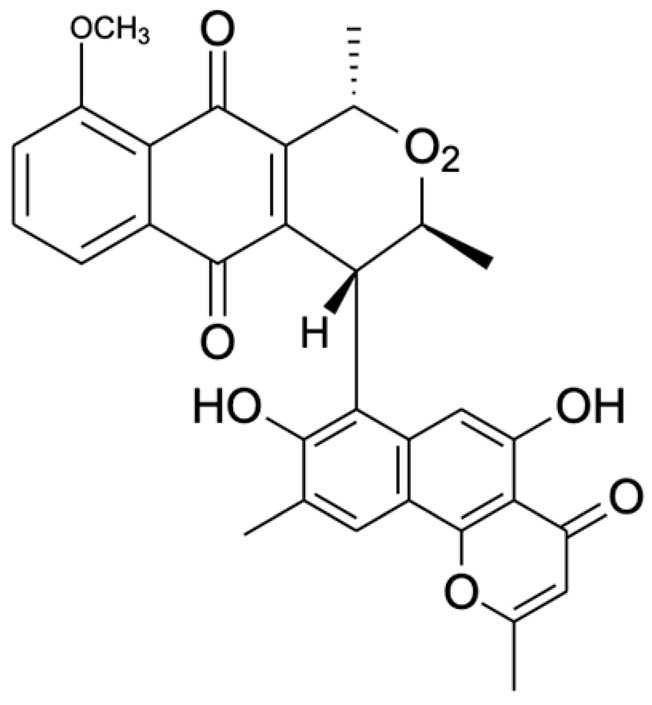	[24]

* IUPAC name for each compound was referred as in PubChem, National Library of Medicine.

## Data Availability

Not applicable.
